# Efficacy of One-Year Treatment with Aflibercept for Diabetic Macular Edema with Practical Protocol

**DOI:** 10.1155/2017/7879691

**Published:** 2017-12-04

**Authors:** Tomomi Kaiho, Toshiyuki Oshitari, Tomoaki Tatsumi, Yoko Takatsuna, Miyuki Arai, Norihiro Shimizu, Eiju Sato, Takayuki Baba, Shuichi Yamamoto

**Affiliations:** Department of Ophthalmology and Visual Science, Chiba University, Graduate School of Medicine, Chiba, Japan

## Abstract

The purpose of this study was to determine the efficacy of one-year treatment of diabetic macular edema (DME) with intravitreal aflibercept (IVA) injections on a practical protocol. The medical records of 51 eyes of 43 patients who were diagnosed with DME and had received IVA treatments were reviewed. The best-corrected visual acuity (BCVA) and the central macular thickness (CMT) were measured at the baseline and at 1, 3, 6, and 12 months after the IVA. The mean number of IVA injections was 3.8 ± 2.4. The mean BCVA was significantly better and the CMT was thinner after the IVA at all follow-up times (*P* < 0.05). The BCVA was better in eyes with a serous retinal detachment (SRD) than without a SRD (*P* < 0.01). There was a significant correlation between the photoreceptor outer segment (PROS) length and BCVA at the baseline and at 12 months after the IVA (*P* < 0.05). A fewer number of IVA injections significantly improved the BCVA and the CMT in eyes with DME after one-year treatment. IVA was more effective in the SRD+ group than in the SRD− group. The PROS length may be a predictive marker for visual outcomes after one-year treatment with IVA for DME (IRB#2272).

## 1. Introduction

Diabetic macular edema (DME) is one of the major causes of moderate vision decrease in patients with nonproliferative diabetic retinopathy [1]. The results of a recent meta-analysis of 22,896 individuals with diabetes (META-EYE Study) found that the prevalence of DME was 6.81% which was comparable to that of proliferative diabetic retinopathy (6.96%) [2]. The results of several clinical trials strongly indicated that repeated injections of vascular endothelial growth factor (VEGF) antibodies improved the visual acuities significantly in eyes with DME [3–10]. On the other hand, the medical cost of frequent anti-VEGF injections is very high which prohibits most patients from receiving frequent injections because of the high costs of the anti-VEGF drugs [11].

We have recently examined real-world data on the efficacy of 6 months of IVA treatments in eyes with DME [12]. The results indicated that a lower number of IVA injections given on a practical protocol significantly improved the visual acuity in eyes with DME [12]. In this one-year study, we examine the efficacy of longer period of IVA injections in eyes with DME. In addition, we compare the efficacy of IVA in eyes with and without a serous retinal detachment (SRD) because the conclusions of the effect of anti-VEGF injections in eyes with or without SRD are still controversial [12–14].

The results of recent studies indicated that the changes in the microstructures of the foveal area are correlated with the visual acuity in eyes with DME [15–20]. For example, Mori et al. suggested that the transverse length of the ellipsoid zone (EZ) of the photoreceptors was significantly correlated with the visual acuity improvement after ranibizumab injections in eyes with DME [15]. Shin et al. demonstrated that the preservations of the EZ and external limiting membrane (ELM) were associated with better visual acuity in eyes with DME [16]. The results of another recent study suggested that the photoreceptor outer segment (PROS) length was significantly correlated with the visual acuity in eyes with DME [17]. Another recent study suggests that the PROS shortening is related to vision loss in eyes with ischemic DME [18]. Shiono et al. indicate that PROS length was a predictive marker of postoperative visual acuities in patients with idiopathic epiretinal membrane [19]. Kogo et al. suggest that PROS length was a predictor of visual outcome in patients with DME after vitrectomy [20]. However, a search of PubMed did not extract any publications that examined the correlation between the PROS length and outer retinal thickness with the visual acuity before and after IVA treatments in eyes with DME.

Thus, the purpose of this study was to determine whether the outer retinal thickness and the PROS length are significantly correlated with the visual acuity at 1-year after IVA treatment in eyes with DME.

## 2. Patients and Methods

The medical records of 51 eyes of 43 consecutive patients who were diagnosed with DME and had received IVA treatments at the Chiba University Hospital from December 2014 to February 2016 were reviewed. Patients with DME who had a reduction of visual acuities and a central macular thickness (CMT) > 250 *μ*m based on the optical coherence tomographic images (SD-OCT, Heidelberg Engineering, Heidelberg, Germany) were included in this study [10]. When the fovea is involved in the edema, patients with focal macular edema were included. Eyes with a CMT < 250 *μ*m, an epiretinal membrane, vitreomacular traction, uveitis, glaucoma, and other retinal diseases and patients with prior brain ischemia or ischemic heart diseases were excluded [10]. In addition, patients who did not agree with the high cost of IVA treatment could not be included in this study. The injection protocol was 1 to 3 consecutive monthly injections, but if the CMT was >300 *μ*m, additional injections were given. If the patients did not agree to the injection, other therapies including vitrectomy and sub-Tenon's capsule triamcinolone acetonide injection were given.

All of the procedures conformed to the tenets of the World Medical Association Declaration of Helsinki. A written informed consent was obtained from all patients and approval for this study was obtained from the Institutional Review Board of the Graduates School of Medicine, Chiba University, Japan (number 2272).

The best-corrected visual acuity (BCVA) and the CMT were measured at the baseline and at 1, 3, 6, and 12 months after the IVA injections. The BCVA was expressed in log⁡MAR (mean ± standard deviation). The presence of a SRD was determined by the detection of subretinal fluid between the retinal pigment epithelium (RPE) and the retina in the optical coherence tomographic images. A SRD was present (SRD+) in 16 eyes and not present (SRD−) in 35 eyes in the IVA group. The patients' data and clinical features are presented in Table 1. Furthermore, the full list of all cases is presented in the supplemental table. Because of the retrospective nature of the study, only eleven patients were treatment-naïve in this study.

Twenty-nine eyes had no disruption of the EZ (EZ+) and 22 eyes had a disruption of the EZ line (EZ−) before the IVA treatment. There were no sight threatening adverse events after the IVA injections.

**Figure 1:**
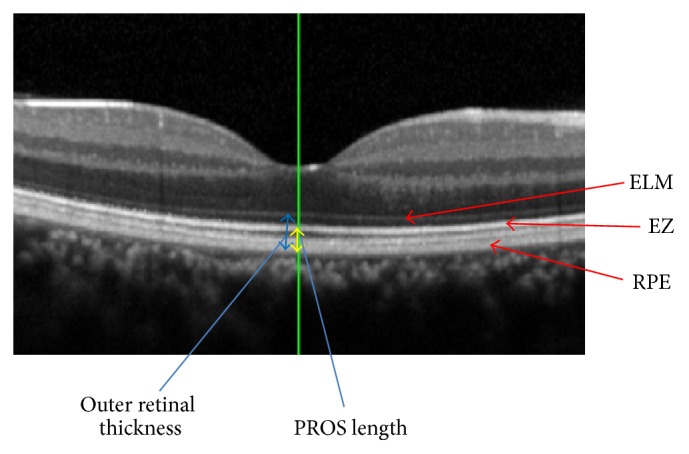
Spectral-domain optical coherence tomographic (SD-OCT) image of a retina showing how the outer retinal thickness and PROS length were measured. The outer retinal thickness was measured from the external limiting membrane (ELM) to the outer border of the highly reflective retinal pigment epithelium (RPE) line. The photoreceptor outer segment (PROS) length was measured from the ellipsoid zone (EZ) to the RPE line.

The PROS length and outer retinal thickness were measured in the cross-sectional OCT images and macular MAP programs. The PROS length was defined as the distance between the EZ line to the highly reflective RPE line. The outer retinal thickness was defined as the distance from the ELM to the RPE line. The outer retinal thickness was automatically measured by the embedded software in the SD-OCT, and the PROS length was measured manually with postprocessing image alignment (Figure 1). The examiner measured the PROS lengths at the fixation point and at 0.5 mm from the fixation point 2 times and the average was used for the statistical analyses. When the foveal center could not detect because of severe macular edema, the fixation points were regarded as the center of the macula. In cases of EZ− group, we did not find patients whose EZ lines were completely disappeared in this study. Thus, we could plot residual EZ lines and drew the provisional lines manually between residual plots to measure PROS length. In eyes with a SRD, the OCT images recorded before the development of a SRD or the first signs of an improvement of the SRD after the IVA treatment were used for the PROS length measurements. Otherwise, the PROS length and outer retinal thickness were measured just before the IVA treatment.

The data are presented as the means ± standard deviations or standard errors. The statistical analyses were performed by Wilcoxon rank test, Mann–Whitney *U*-test, repeated measured analysis of variance (ANOVA), and Spearman rank correlation with Stat View 5.0 software. A *P* < 0.05 was considered significant.

## 3. Results

**Figure 2:**
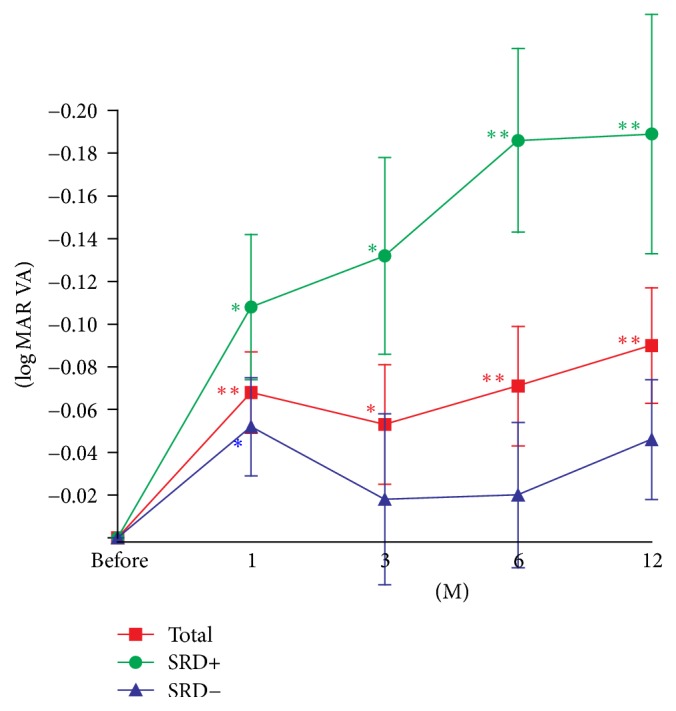
Changes in the mean best-corrected visual acuity (BCVA) expressed in logarithm of the minimum angle of resolution (log⁡MAR) units before and after an intravitreal aflibercept (IVA) injection in eyes with a serous retinal detachment (SRD+) or without a SRD (SRD−). Data are expressed as the means ± standard error of the means (SEMs). ^*∗*^*P* < 0.05; ^*∗∗*^*P* < 0.01 relative to the baseline best-corrected visual acuity (BCVA).

The BCVAs were significantly better at 1, 3, 6, and 12 months than at the baseline after the IVA injections (*P* = 0.0008, 0.0144, 0.0035, and 0.0013, resp.; Figure 2, Table 2). In the SRD+ group, the BCVAs were significantly better at 1, 3, 6, and 12 months than at the baseline after the IVA injections (*P* = 0.0176, 0.0166, 0.0021, and 0.0075, resp.; Figure 2). In the SRD− group, the BCVAs were significantly better only at 1 month after the IVA injections (*P* = 0.0227; Figure 2). Repeated measured ANOVA showed a significant difference in the BCVA between the SRD+ and SRD− groups (*P* = 0.0041). Thus, the BCVAs improved more significantly in the SRD+ group than in the SRD− group after the IVA injections.

**Figure 3:**
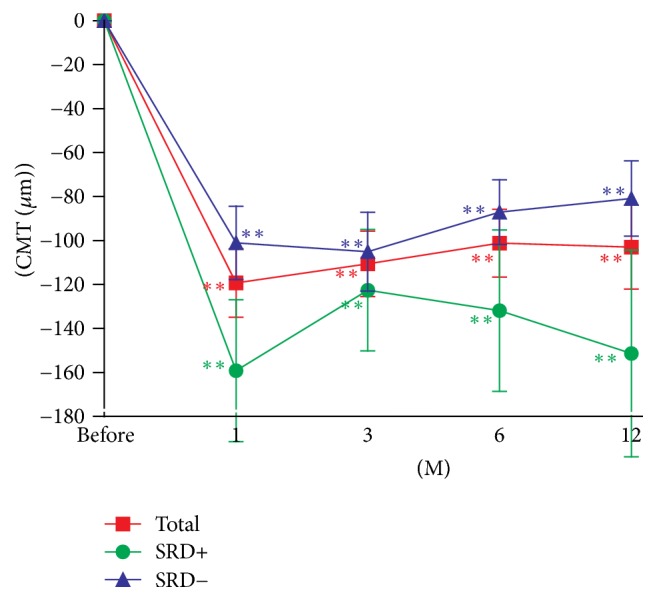
Changes of the mean central macular thickness (CMT) before and after IVA injections in eyes with or without a SRD. Data are expressed as the means ± SEMs. ^*∗∗*^*P* < 0.01 relative to the baseline CMT.

The mean CMT was significantly reduced at 1, 3, 6, and 12 months from that at the baseline after the IVA injections (*P* < 0.0001 for all; Figure 3, Table 3). In the SRD+ group, the mean CMT was significantly reduced at 1, 3, 6, and 12 months after the IVA (*P* = 0.0009, 0.0016, 0.0052, and 0.0097, resp.; Figure 3). In the SRD− group, the mean CMT was also significantly reduced at 1, 3, 6, and 12 months after the IVA (*P* < 0.0001 for all; Figure 3). Repeated measured ANOVA showed that the difference in the CMT between the SRD+ and SRD− groups was not significant (*P* = 0.0914).

The coefficient of correlation between outer retinal thickness and PROS length was significant at the baseline (*ρ* = 0.374; *P* = 0.0086; *n* = 51). The coefficients of correlation between the PROS length before the IVA injections and the BCVAs before the IVA injections and the BCVAs 12 months after the IVA were significant (*ρ* = −0.281, *P* = 0.0399, *n* = 51; *ρ* = −0.321, *P* = 0.0192, *n* = 51, resp.). Thus, the PROS length before the IVA injections was significantly correlated with the BCVAs at 12 months after the IVA injections. On the other hand, the correlation between the outer retinal thickness before the IVA injections and the BCVAs before and 12 months after the IVA were not significant (*ρ* = −0.109, *P* = 0.3969, *n* = 51; *ρ* = −0.095, *P* = 0.4558, *n* = 51, resp.). Thus, the outer retinal thickness before the injections was not significantly correlated with the BCVAs at 12 months after the IVA injections. The correlations between the PROS length and the outer retinal thickness and CMT before the IVA injections were not significant (*ρ* = −0.031, *P* = 0.8180, *n* = 51; *ρ* = −0.128, *P* = 0.3521, *n* = 51, resp.). Thus, the PROS length and outer retinal thickness were not correlated with the CMT.

The comparisons among the values of the parameters in the EZ+ group and the EZ− group are shown in Table 4. In the EZ+ group, the BCVA before the IVA treatments was significantly better than in the EZ− group (*P* < 0.0001; Table 4). In addition, the BCVA at 12 months after the IVA injections was significantly better in the EZ+ group than in the EZ− group (*P* = 0.0492; Table 4). In the EZ+ group, the mean PROS length was significantly longer than in the EZ− group (*P* = 0.0003; Table 4). Thus, the presence or absence of a disrupted EZ was associated with a longer or shorter PROS length.

## 4. Discussion

The results of several real-world studies on the effect of anti-VEGF therapies over a 1-year period have been reported in eyes with DME [21–25]. However, most of the results were obtained from that after intravitreal ranibizumab (IVR) injections [21–25]. As best we know, our study is the first real-world study of the effects of 1-year IVA treatments on eyes with DME. Compared to the ideal world evidence of clinical trials [7, 8], the mean numbers of anti-VEGF injections were fewer, and the efficacy was lower in the real-world evidence of anti-VEGF treatment for DME. In the VISTA and VIVID studies [8], the mean number of IVA injections was 9 to 12 times/year, and in the REVEAL study [7], the mean number of ranibizumab injections was 7 to 8 times/year. On the other hand, the mean number of ranibizumab injections was 7 times/year in a practical study in the United Kingdom [21]. In studies in Denmark [22] and France [23], the mean numbers of ranibizumab injections were 5 times/year. In the United States [24], the mean number of anti-VEGF injections was 5.8 for the first year. In a study in Italy [25], the mean number of IVR injections was only 4 for a period of 18 months. In our study, the mean number of IVA injections was less than 4 times/year. Nevertheless, the BCVAs after 1 year of IVA were significantly improved compared to that at the baseline (Figure 2). Thus, even with less frequent injections, the results of IVA may be comparable to that of the VISTA and VIVID studies [8]. The lower number of IVA injections indicates that it would be more cost effective than the higher numbers obtained from the clinical trials.

Our results indicate that the BCVAs in eyes with DME and a SRD were improved more significantly than in eyes with DME without a SRD. This is consistent with our earlier short-term study [12] and also with Seo et al. who reported that eyes with DME and SRD required more frequent ranibizumab injections than eyes with DME with diffuse retinal thickening [13]. In our study, however, the mean number of injections in eyes with DME and a SRD was 3.9 ± 3.0 times/year and without a SRD was 3.7 ± 2.1 times/year (*P* = 0.8526; Mann–Whitney *U*-test). The results of our study are consistent with a recent study that reported better visual improvement in eyes with DME with a SRD [14]. However, we cannot eliminate the possibility that the visual improvement may have been associated with a lower baseline BCVA. In this study, the baseline mean BCVAs in eyes with DME with SRD was 0.56 ± 0.34log⁡MAR units and without SRD was 0.32 ± 0.25log⁡MAR units (*P* = 0.0135, Mann–Whitney *U*-test). Although VEGF may have been accumulated in the fluid of the SRD and anti-VEGF agents may reduce the activity of the accumulated VEGF in the subretinal space [12], additional studies are needed to determine the efficacy of IVA in DME with and without SRD.

The PROS length is defined as the distance between the EZ and the RPE line, and the length ranges from 25 *μ*m to 63 *μ*m in the macula area in histological measurements in humans [26, 27]. In our cohort, the mean PROS length was 57.8 ± 10.0 *μ*m which is comparable to the histological measurements [26, 27]. Our results indicated that the PROS length before treatment was significantly correlated with the BCVAs 12 months after IVA treatment. This means that shorter PROS lengths are correlated with poorer visual acuities. In addition, our findings showed that a shorter PROS length was associated with a disruption of the EZ line (Table 4). The EZ is useful for evaluating the integrity of the foveal photoreceptor and is significantly correlated with the final visual acuity in eyes with DME [15, 16]. A recent study suggests that PROS length is a predictive factor for the visual outcome after anti-VEGF injections for eyes with retinal vein occlusion [28]. Our results showed that the PROS length can be used as a predictive marker for the visual outcome at 12 months after IVA injections in eyes with DME. On the other hand, the outer retinal thickness is not correlated with the visual acuity at any time during the study. The outer retinal thickness includes not only the PROS length but also the nuclei and axons of the photoreceptors. Thus, changes of PROS length could be masked by the other cellular components in the outer retinal thickness.

CMT was not correlated with neither visual acuities nor PROS length. In some cases, the changes of CMT are not paralleled with the changes of BCVAs. For example, CMT was decreasing in the SRD− group at 3 months after IVA injection compared with 1 month after IVA, but the BCVAs were worsening. That is why we focused on the microstructures of the fovea for the evaluation of visual function in eyes with DME in this study. Our results suggest that only the outer segments of the photoreceptors are important for predicting the visual acuity after IVA treatment in eyes with DME.

This study has limitations. This was a retrospective study on a small number of eyes. In addition, not all patients were treatment-naïve because of the retrospective nature of this study. Thus, the conclusion of this study should be interpreted with caution. Further large prospective studies are needed to examine the efficacy of IVA in eyes with DME.

In conclusion, a lower number of IVA injections can significantly improve the BCVA and reduce the CMT in eyes with DME. The effectiveness of IVA is not dependent on the presence or absence of a SRD. The PROS length is a predictive factor for visual outcome 12 months after IVA therapy in eyes with DME.

## Figures and Tables

**Table 1:** Clinical data and features.

Age (years)	64.7 ± 10.8
Gender (men : women)	27 : 24
HbA1c (%)	7.8 ± 1.7
DM duration (years)	8.6 ± 9.5
BCVA (log⁡MAR units before)	0.39 ± 0.30
CMT (*μ*m; before)	489.6 ± 106.8
Injection times	3.8 ± 2.4
SRD (SRD+ : SRD−)	16 : 35
Pretreatment of	
IVR/IVB (eyes)	27
PC (eyes)	34
STTA (eyes)	38
Additional treatment	STTA 3, PC 5, PPV 1
Length from RPE to EZ line (*μ*m)	57.8 ± 10.0
Length from RPE to ELM line (*μ*m)	82.6 ± 6.3

BCVA, best-corrected visual acuity; CMT, central macular thickness; SRD, serous retinal detachment; IVR, intravitreal ranibizumab injection; IVB, intravitreal bevacizumab injection; PC, photocoagulation; STTA, sub-Tenon's capsule triamcinolone acetonide; RPE, retinal pigment epithelium; EZ, ellipsoid zone; ELM, external limiting membrane.

**Table 2:** The real values of BCVA (log⁡MAR VA) before and after IVA treatment in eyes with (SRD+) and without (SRD−) a serous retinal detachment.

Before IVA (total)	0.39 ± 0.30
1 M after IVA	0.32 ± 0.29
3 M after IVA	0.34 ± 0.33
6 M after IVA	0.32 ± 0.31
12 M after IVA	0.30 ± 0.28

Before IVA (SRD+)	0.53 ± 0.33
1 M after IVA	0.43 ± 0.34
3 M after IVA	0.39 ± 0.36
6 M after IVA	0.36 ± 0.34
12 M after IVA	0.36 ± 0.30

Before IVA (SRD−)	0.32 ± 0.25
1 M after IVA	0.27 ± 0.25
3 M after IVA	0.30 ± 0.31
6 M after IVA	0.30 ± 0.28
12 M after IVA	0.28 ± 0.27

BCVA, best-corrected visual acuity; IVA, intravitreal aflibercept injection.

**Table 3:** The real values of CMT before and after IVA treatment in eyes with (SRD+) and without (SRD−) a serous retinal detachment.

Before IVA (*μ*m; total)	489.6 ± 106.8
1 M after IVA	370.3 ± 96.1
3 M after IVA	379.0 ± 106.4
6 M after IVA	388.4 ± 113.1
12 M after IVA	386.6 ± 116.5

Before IVA (*μ*m; SRD+)	536.3 ± 109.4
1 M after IVA	386.0 ± 136.1
3 M after IVA	413.4 ± 130.1
6 M after IVA	417.2 ± 148.0
12 M after IVA	387.6 ± 159.3

Before IVA (*μ*m; SRD−)	464.1 ± 95.0
1 M after IVA	363.0 ± 64.7
3 M after IVA	359.0 ± 86.4
6 M after IVA	376.9 ± 87.1
12 M after IVA	383.2 ± 88.8

CMT, central macular thickness; IVA, intravitreal aflibercept injection.

**Table 4:** Comparisons of parameters in EZ+ and EZ− eyes with DME.

	EZ+	EZ−	*P* values
BCVA (log⁡MAR units; before)	0.24 ± 0.20	0.60 ± 0.28	**P** < 0.0001
BCVA 12 M after IVA (log⁡MAR units)	0.15 ± 0.20	0.25 ± 0.21	**P** = 0.0492
CMT (*μ*m; before)	466 ± 107	521 ± 100	*P* = 0.0705
CMT 12 M after IVA (*μ*m)	365 ± 81	415 ± 149	*P* = 0.3134
Mean PROS length (*μ*m)	62.1 ± 8.3	52.1 ± 9.5	**P** = 0.0003
Mean outer retinal thickness (*μ*m)	82.9 ± 4.5	82.1 ± 8.2	*P* = 0.4285

EZ, ellipsoid zone; BCVA, best-corrected visual acuity; CMT, central macular thickness; IVA, intravitreal aflibercept injection; PROS, photoreceptor outer segment.

## Supplementary Materials

Supplementary Materials Supplemental Table. A list of all cases in this study.  
