# Supplementary material for: Efficacy of One-Year Treatment with Aflibercept for Diabetic Macular Edema with Practical Protocol
Source: Biomed Res Int. 2017 Dec 4;2017:7879691. doi: 10.1155/2017/7879691 (PMC5733831; doi:10.1155/2017/7879691)
Supplement: Supplementary Materials — Supplemental Table. A list of all cases in this study. [file 7879691.f1.pdf]

sex	age	right/left	IVA injection time	history of IVR/IVB	0 M logMAR BCVA	1Y changes of logMAR BCVA	0 M CMT	1Y changes of CMT	SRD	EZ	history of PC	history of STTA	cataract/IOL	Additional treatment
female	66	right	1	IVR 1 time	0.301029996	0	297	187	+	+	+	+	cat	-
female	66	left	8	IVR 3 times	1	-0.301029996	629	-325	+	+	+	+	cat	-
female	52	right	3	IVR 3 times	0.397940009	0.425968732	561	162	+	-	+	+	cat	PC
male	42	left	12	IVR 9 times	0.698970004	-0.602059991	400	-84	+	-	-	+	clear	-
female	75	right	3	IVR 1 time	0.823908741	-0.124938737	560	-168	+	-	+	+	IOL	STTA
female	67	left	7	IVR 3 times	0.522878745	-0.301029996	491	143	+	-	+	+	IOL	-
female	64	left	1	IVR 2 times	1.301029996	-0.477121255	788	-147	+	-	+	+	cat	-
male	61	right	4	IVR 2 times	0.301029996	-0.079181246	532	-245	+	-	+	+	cat	-
male	52	right	4	IVR 1 time	0.15490196	-0.15490196	551	-159	+	+	-	-	cat	-
female	54	left	5	IVB 6 times	1	-0.301029996	646	-438	+	-	+	+	cat	-
male	70	right	3	-	0.397940009	-0.301029996	570	-315	+	-	+	+	IOL	PPV
male	42	left	1	-	0.22184875	-0.22184875	627.8	-353.6	+	+	+	+	clear	-
female	73	right	1	-	0.522878745	-0.124938737	483	-256	+	-	+	-	IOL	-
male	72	left	2	-	0.15490196	-0.109144469	426	-179	+	+	+	+	IOL	-
male	71	right	3	-	0.698970004	-0.176091259	577	-12	+	-	-	-	IOL	-
male	71	left	5	-	0.397940009	-0.176091259	589	-233	+	+	-	-	IOL	-
male	67	left	6	IVR 2 times	0.698970004	-0.301029996	480	-148	-	-	+	+	IOL	-
female	64	right	1	IVR 4 times	0.823908741	-0.124938737	446	-24	-	-	+	+	cat	-
female	65	left	3	IVB 1 time	0.22184875	-0.176091259	388	74	-	+	-	+	IOL	-

female	70	right	4	IVB 1 time	0.301029996	0	346	-11	-	+	+	+	cat	-
female	54	right	3	IVR 1 time	0.096910013	-0.096910013	263	158.8	-	+	+	+	cat	-
male	73	left	3	IVR 1 time	0.096910013	-0.096910013	448	-22	-	+	-	+	IOL	-
male	42	right	8	IVR 7 times	-0.079181246	0	358	59	-	+	-	+	clear	-
male	83	right	2	IVR 3 times	0.698970004	0	678	-52	-	-	+	+	IOL	-
male	81	right	1	IVR 1 time	0.301029996	0.096910013	401	-96.2	-	+	+	-	IOL	-
female	68	right	6	IVR 3 times	0.301029996	-0.380211242	419	-121	-	+	+	+	cat	-
female	62	right	8	IVR 4 times	0	-0.079181246	607	-281	-	+	-	+	cat	-
female	62	left	7	IVR 3 times	0.15490196	0.06694679	436	-187	-	+	-	+	cat	-
male	76	left	6	IVR 5 times	0.301029996	-0.146128036	642	-107	-	+	+	+	IOL	-
male	61	left	5	IVR 2 times	0.522878745	-0.22184875	472	-122	-	-	+	+	cat	-
male	54	right	5	IVB/IVR<br>1 time respectively	0.301029996	0.397940009	540	-163	-	-	+	+	cat	-
male	68	right	3	IVR 3 times	0.301029996	0.096910013	546	17	-	+	+	+	cat	-
male	56	right	3	IVR 1 times	0.397940009	-0.301029996	485	-93	-	+	-	+	cat	-
male	64	left	5	-	0.22184875	0	433	-50	-	-	+	-	IOL	PC
male	83	left	4	-	0.522878745	0.176091259	531	22	-	-	+	+	IOL	-
female	65	left	1	-	0.045757491	-0.045757491	436	-141	-	+	-	-	IOL	-
male	56	left	7	-	0.15490196	0.06694679	494	-130	-	+	+	+	IOL	-
female	76	right	4	-	0.823908741	-0.124938737	320	22	-	-	+	+	IOL	PC

male	69	left	1	-	0.698970004	-0.176091259	587	-296	-	+	+	+	cat	-
female	69	right	3	-	0.397940009	-0.176091259	376	-75	-	+	-	-	cat	-
female	58	left	3	-	0.301029996	-0.146128036	592	-245	-	+	-	+	cat	-
female	69	left	5	-	0.522878745	0	477	-232	-	-	+	+	cat	-
male	44	right	4	-	0	0	384	-71	-	+	-	-	cat	STTA and PC
male	44	left	4	-	0.15490196	0.243038049	568	-174	-	+	-	-	cat	STTA and PC
female	73	right	3	-	0.823908741	0	421	-45	-	-	+	+	cat	-
male	68	left	3	-	0.096910013	0.204119983	562	-88	-	-	+	-	cat	-
female	78	left	1	-	0.397940009	0.124938737	486	-80	-	-	+	+	IOL	-
female	58	right	4	-	0.15490196	-0.109144469	339	1	-	+	-	+	cat	-
male	75	right	2	-	0.22184875	-0.124938737	415	46	-	+	-	+	IOL	-
female	72	right	1	-	0.22184875	-0.22184875	417	-100	-	+	+	-	IOL	-
male	75	left	1	-	0	0	450	-76	-	+	+	-	IOL	-

Supplemental Table. A list of all cases in this study.

Abbreviations: IVA, intravitreal aflibercept injection; IVR, intravitreal ranibizumab injection; IVB, intravitreal bevacizumab injection, BCVA, best-corrected visual acuity; CMT, central macular thickness; SRD, serous retinal detachment; EZ, ellipsoid zone, PC, photocoagulation; STTA, sub-Tenon's triamcinolone acetonide; cat, cataract; PPV, pars plana vitrectomy; IOL, intraocular lens.
